# Detecting the existence of an invisibility cloak using temporal steering

**DOI:** 10.1038/srep15571

**Published:** 2015-10-23

**Authors:** Shin-Liang Chen, Ching-Shiang Chao, Yueh-Nan Chen

**Affiliations:** 1 Department of Physics and National Center for Theoretical Sciences, National Cheng Kung University, Tainan 701, Taiwan

## Abstract

An invisibility cloak provides a way to hide an object under the detection of waves. A good cloak guides the incident waves through the cloaking shell with few distortion. Even if one day a nearly perfect cloak is built, some important quantum effects, such as dephasing of the electron spin or photon polarization, may still remain. In this work, we consider the possibility that using the temporal steering of these degrees of freedom to detect the existence of an invisibility cloak.

Invisibility cloak based on the transformation design method (TDM) has attracted great attentions in the past decade^1^,^2^,^3^,^4^,^5^,^6^,^7^,^8^,^9^,^10^,^11^,^12^,^13^,^14^,^15^,^16^,^17^,^18^. The main idea of the TDM is to perform the coordinate transformation on the wave equation of the corresponding cloaking wave to create the hiding region (Fig. 1). To keep the form of the equation invariant, the metric tensors are combined with the specific parameters, which are usually the properties of the material of the cloaking shell. For instance, the TDM for electromagnetic waves^1^,^2^,^3^ reinterprets the effect of the coordinate transformation in conductivity and permeability of the original non-transformed system. Similarly, cloaking of matter waves^4^,^5^,^6^,^7^,^8^,^19^,^20^,^21^,^22^,^23^ requires a proper design of the effective mass and potential of the cloaking shell. There are also other kinds of cloak, such as cloaking of elastic waves^9^,^10^,^11^,^12^,^13^,^14^, liquid waves^15^,^16^, heat flows^17^,^18^, etc. Waves incident onto the cloak designed by the TDM are guided through the cloaking shell without any scattering and distortion.

Einstein-Podolsky-Rosen (EPR) steering^24^,^25^,^26^,^27^ is one of the quantum correlations that allows one party to remotely prepare some specific states for the other party via choosing different measurement settings. The degree of the non-locality of EPR steering is stronger than the entanglement but weaker than the Bell non-locality^26^. EPR steering can be verified via the steering inequalities^27^, which are built on the fact that the correlations cannot be explained by the local hidden state model. Apart from the correlations between two (or more) parties, quantum correlations may also occur in single party at different times. For example, Leggett and Garg derived an inequality^28^,^29^ under the assumption of macroscopic realism and non-invasive measurement. It can be used to verify the quantum coherence of a macroscopic system under the weak measurements^30^. Recently, a temporal analog of the steering inequality—the temporal steering inequality^31^—also focuses on the correlations of a single party at different times. Moreover, the classical bound of temporal steering inequality is found to have deep connection with the quantum cryptography.

Motivated by these developments, we ask the question: how to crack an invisibility cloak when a nearly perfect cloak is built? Given the fact that some quntum effect, such as dephasing, is almost inevitable for the waves passing through a material, we consider the possibility that using the temporal steering to crack the invisibility cloak. For concreteness, we consider the invisibility cloak of the electromagnetic waves and the electron matter waves. We assume the polarizations of the incident electromagnetic waves suffer a phase damping when passing through the cloaking shell. Secondly, since the spin of the incident matter waves (e.g., an electron) may interact with the hiding object when passing through the cloaking shell, we assume the incident particle experiences a coherent coupling. The feature of temporal steering inequality is that the temporal steering parameter always maintains the maximal value if the wave does not interact with other ancillary systems (or environment). Our results show that the temporal steering parameter of the incident waves varies with the traveling time in the cloaking shell, and therefore the temporal steering may be used to crack the invisibility cloak.

## Results

### Transformation design method for waves

One of the crucial points in the TDM for waves is to perform the appropriate coordinate transformation on the spatial (time-independent) wave equation from the coordinate system *q* to 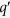, and keep the form invariant^1^,^7^,^8^


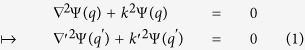


where 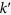 reinterprets the effect of the coordinate transformation in the material properties of the cloaking shell.

**Figure 2:**
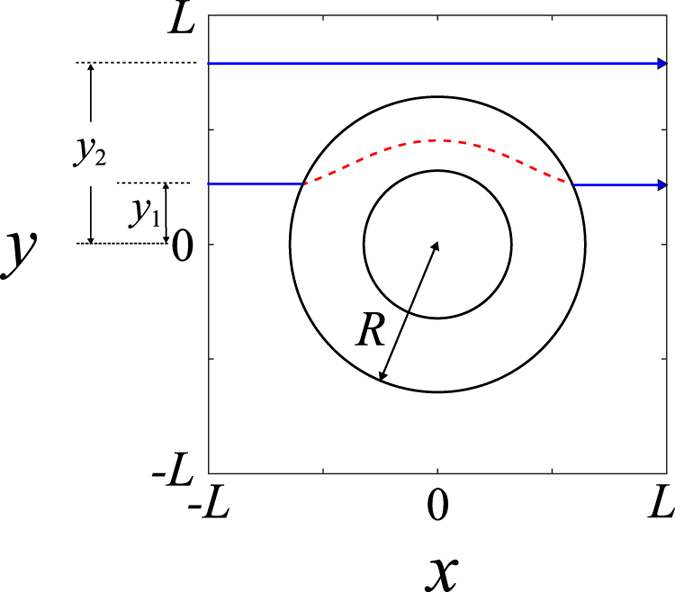
The trajectory of the incident particle. The trajectory of the incident particle (photons or electrons) from the current density in the classical limit. The direction of the incident plane waves 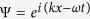 is along +*x*.

The behavior of the incident waves can be visualized through the current density **J**, with the continuity equation


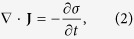


where 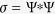 is the probability density of the wave function. The incident plane wave 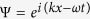 could be the electromagnetic wave (photons) or the matter wave (electrons). One can use the relation, 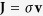, to obtain the classical trajectory of the incident particle^32^. In classical limit, the velocity vector **v** is tangent to the particle trajectory. Therefore, the trajectory of the incident waves can be obtained from the current density (Fig. 2).

Moreover, it is necessary to estimate the time interval *t*_s_ of the incident particle staying inside the cloaking shell. The phase of the incident wave after passing through the shell of a perfect cloak should be the same as that traveling in free space. Thus, the time intervals for different trajectories should be the same. As an example, we consider two trajectories, representing the path that the particles travel from *x* = −*L* to *x* = +*L* with and without passing through the shell, respectively (Fig. 2). The time interval *t*_s_ can then be easily obtained


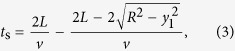


where 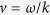 is the velocity of the incident particle outside the cloak.

### Temporal steering inequality

Now, we briefly describe the concept of the temporal steering inequality^31^. Consider a two-level system sent into one of the channels λ with the probability *q*_λ_. During the transmission, there are two observers, Alice and Bob. Firstly, Alice performs the measurement on the system at time *t*_A_ along the basis *i* with the outcomes 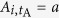. Then, the system is suffered from the influence of the channel for a time interval before Bob receives it. When Bob receives the system at time *t*_B_, he obtains the outcomes 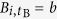 by performing the measurement along the same setting *i*. If Alice's choice of measurement has no influence on the state that Bob receives, the following temporal steering inequality holds


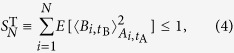


and the bound that quantum mechanics gives is


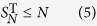


where *N*(=2 or 3) is the number of the mutually unbiased measurements that Bob implements on the system, and


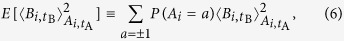


with


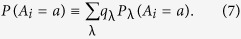


Here, Bob's expectation value conditioned on Alice’s result is defined as


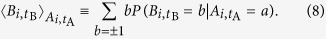


**Figure 3:**
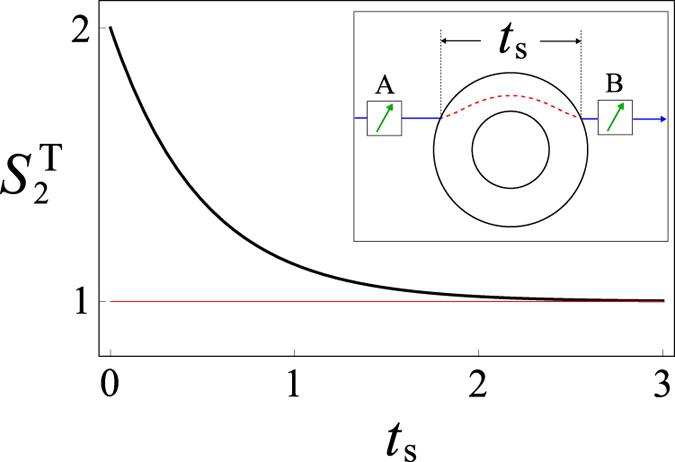
The dynamics of the temporal steering parameter 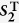 of the polarizations of the incident photons when suffering a phase damping inside the cloaking shell. The horizontal red line represents the classical bound of the temporal steering inequality. In plotting the figure, the time *t*_s_ is in units of 1/*γ*. Inset: The schematic diagram of the time interval that the temporal steering inequality is applied when the incident particle passes through the cloak.

Here, we would like to use two measurement settings, the 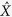 and 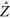 bases, rather than three. Since three measurement settings are sufficient to perform the quantum state tomography, using the temporal steering inequality thus requires fewer resources. We use one of the features of the temporal steering parameter 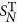 in equation (4) to detect the quantum cloak (inset of Fig. 3): If the system does not suffer any interaction, quantum mechanics predicts that 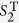 always maintains the maximal value 2. If 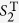 varies with time, the system is subject to some dynamics.

#### Cracking electromagnetic cloak by using the temporal steering.

We assume the incident photons suffer a phase damping with decay rate γ when traveling through the cloaking shell. The state of the polarizations can be described by the density matrix 
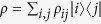
, where 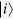, 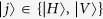 with 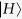 and 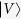 being the horizontal and vertical polarizations, respectively. The initial state is prepared in the maximally mixed state


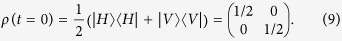


The evolution of the polarizations inside the cloaking shell can be obtained by solving the following Markovian master equation with Lindblad form^33^,^34^


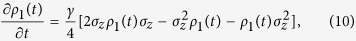


where *σ*_*z*_ is the Pauli-*z* matrix. From equations (4), (9), and (10) the steering parameter can be obtained straightforwardly


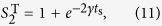


where *t*_s_ is defined in equation (3). Here, the two bases are 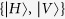 and 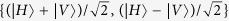. The dynamics of the temporal steering parameter 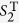 of the polarizations is plotted in Fig. 3. We can see that the temporal steering parameter 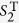 varies with time inside the shell (*t*_s_). Therefore, the electromagnetic cloak is cracked by using the temporal steering.

#### Cracking quantum cloak by using the temporal steering

In this section, we use the temporal steering parameter to detect the dynamics of the spin of a quantum particle inside the cloaking shell. For simplicity, we consider the incident matter wave with the spin-1/2 degree of freedom, e.g. electrons. We further assume the spin of the electron experiences the coherent coupling from the ancillary spin hidden inside the cloaking shell. The state of the incident spin can be described as 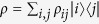, where 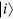, 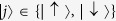 with 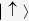 and 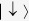 being the spin-up and spin-down state, respectively. The interaction Hamiltonian can be written as 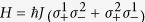, where 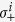 and 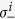 are the raising and lowering operators of the *i* th spin, and 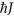 is the coupling strength. The evolution of the entire system inside the cloaking shell can be obtained by the quantum Liouville equation


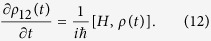


The state of the incident electron *ρ*_1_(*t*) can be obtained by tracing out the ancillary electron, i.e. 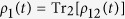. We choose the initial state as


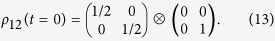


**Figure 4:**
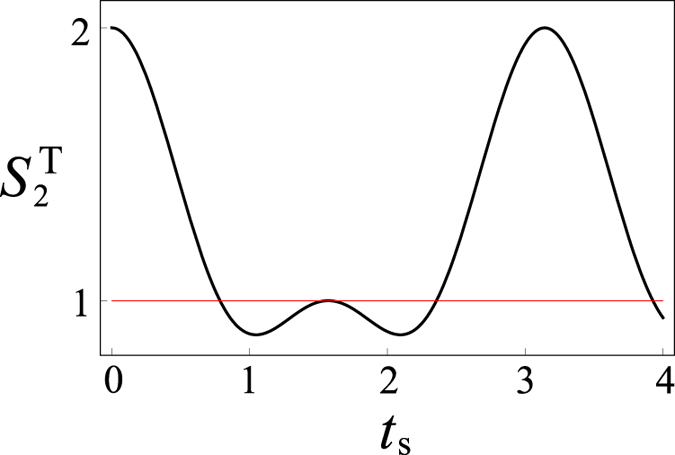
The dynamics of the temporal steering parameter 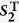 of the spin of the incident electron when it passes through the cloaking shell. The horizontal red line represents the classical bound of the temporal steering inequality. In plotting the figure, the time *t*_s_ is in units of coupling strength *J*, and 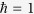.

The temporal steering parameter 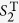 is then written as


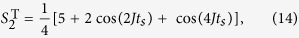


where the two bases are Pauli 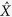 and 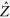. From Fig. 4, we can see that 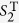 varies with time, indicating the incident electron is influenced by the hiding spin. Therefore, the quantum cloak is cracked by using the temporal steering.

## Discussion

One may notice that there are other ways to crack the electromagnetic cloak. For example, using the classical polarized fields, one can measure the alternation of the coherence due to the dephasing effect. Besides, if the electromagnetic cloak is designed within a finite frequency range, the cloak can be easily detected by using the electromagnetic waves with the frequency outside that range. To crack the quantum cloak, a simple way is to detect whether the direction of the spin is changed. However, this method requires the measurement direction of the receiver to be synchronized with that of the sender. In the temporal steering scenario, there is no such constraint, i.e. the steering inequality still holds even if the bases are not synchronized^31^. Another way to crack the quantum cloak is the quantum state tomography. In this case, one has to use three bases (for qubit system) to perform the tomography, whereas one only needs two bases for the temporal steering inequality. One may also use the degree of entanglement to detect the cloak: preparing initially the entangled pair, sending one of them into the shell, and measuring the degradation of the entanglement. In conclusion, the temporal steering provides one of the feasible ways to crack both the electromagnetic and quantum cloak.

## Additional Information

**How to cite this article**: Chen, S.-L. *et al.* Detecting the existence of an invisibility cloak using temporal steering. *Sci. Rep.* **5**, 15571; doi: 10.1038/srep15571 (2015).

## Figures and Tables

**Figure 1:**
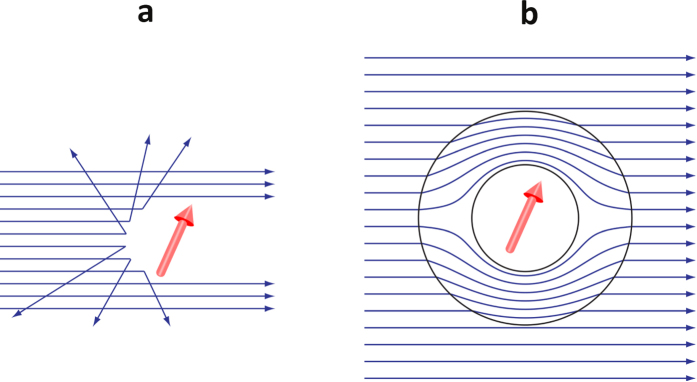
Schematic diagram of invisibility cloak. (**a**) An object is observed by the detection of the scattering waves. (**b**) A cloak designed by the TDM perfectly guides the incident waves passing through the cloaking shell.
